# Sudden cardiac death caused by myocarditis in persons aged 1–49 years: a nationwide study of 14 294 deaths in Denmark

**DOI:** 10.1080/20961790.2019.1595352

**Published:** 2019-08-19

**Authors:** Thomas Hadberg Lynge, Trine Skov Nielsen, Bo Gregers Winkel, Jacob Tfelt-Hansen, Jytte Banner

**Affiliations:** a Department of Cardiology, Rigshospitalet, Denmark;; b Department of Forensic Medicine, Section of Forensic Pathology, Aarhus University, Aarhus, Denmark;; c Department of Forensic Medicine, Section of Forensic Pathology, Copenhagen University, ‎Copenhagen, Denmark

**Keywords:** Forensic sciences, sudden cardiac death, myocarditis, autopsy, epidemiology, gender, children, young adults

## Abstract

Myocarditis is associated with an increased risk of sudden cardiac death (SCD) in the young. However, information on nationwide burden of SCD caused by myocarditis (SCD-myocarditis) is sparse. For this study all deaths among persons in Denmark aged 1–35 years in 2000–2009 and 36–49 years in 2007–2009 (27.1 million person-years) were included. Autopsy reports, death certificates, discharge summaries, and nationwide registries were used to identify all cases of SCD-myocarditis. In the 10-year study period, there were 14 294 deaths, of which we identified 1 363 (10%) SCD. Among autopsied SCD (*n* = 753, 55%), cause of death was myocarditis in 42 (6%) cases corresponding to an SCD-myocarditis incidence of 0.16 (95%CI: 0.11–0.21) per 100 000 person-years. Males had significantly higher incidence rates of SCD-myocarditis compared to females with an incidence rate ratio of 2.2 (95%CI: 1.1–4.1). Myocarditis was not registered as cause of death in any of the non-autopsied SCD (*n* = 610, 45%). In conclusion, after nationwide unselected inclusion of 14 294 deaths, we found that 6% of all autopsied SCD was caused by myocarditis. No cases of SCD-myocarditis were reported in the non-autopsied SCD, which could reflect underdiagnosing of myocarditis in non-autopsied SCD. Furthermore, our data suggest a female protection towards SCD-myocarditis.

## Introduction

Myocarditis refers to an inflammatory disorder of the myocardium and is characterized by infiltration of immunocompetent cells and non-ischemic degeneration of cardiac myocytes [1,2]. Myocarditis can result from a wide spectrum of causes, including infectious pathogens, toxins, and hypersensitivity reactions with viral infections reported as the most common cause of myocarditis in western countries [1,3]. The clinical presentation of myocarditis is highly variable ranging from subclinical disease to acute or slowly progressing heart failure or sudden cardiac death (SCD) [1,2]. The clinical diversity at presentation, which is mainly dominated by nonspecific symptoms and findings, complicates the diagnosis of myocarditis [4–6]. Different approaches for the diagnosis of myocarditis exist. Several noninvasive diagnostic tests, such as serum biomarkers, electrocardiography, echocardiography, and cardiovascular magnetic resonance are available, but the results are often nonspecific [1]. Histological cardiac examination remains the gold standard for an unequivocal diagnosis of myocarditis and in 1995, the World Health Organization/International Society and Federation of Cardiology Task Force defined myocarditis as an inflammatory disease of the heart muscle based on histological, immunological, and immunohistochemical criteria [3,7]. Despite this rather clear-cut definition, the diagnosis of myocarditis continues to prompt considerable debate, particularly because the clinical and histological diagnoses are poorly correlated [6,8,9].

Myocarditis has been reported to be a major cause of sudden and unexpected death in infants, adolescents, and young adults. However, the proportion of SCD caused by myocarditis (SCD-myocarditis) has been variably reported ranging from 1%–14% among the young [10–16]. These differences are likely explained by inhomogeneity in the study populations, as well as differences in SCD definition and classification/definition of myocarditis postmortem.

We have previously in a nationwide and unselected setting used autopsy reports, deaths certificates, and information from national registries to identify all SCD in Denmark in persons aged 1–35 years in 2000–2009 and 36–49 years in 2007–2009 [10,11]. The aim of the present study was to assess incidence of SCD-myocarditis in the young Danish population using well-defined SCD cases.

## Materials and methods

This nationwide population-based study conducted in Denmark covers all deaths among individuals aged 1–35 years in 2000–2009 and 36–49 years in 2007–2009 [10,11]. Autopsy reports, death certificates, and information from national registries were used to identify and characterize all cases of SCD caused by myocarditis.

### The Danish healthcare system and Danish registries

The Danish National Health service provides tax-financed public healthcare for all Danish residents free of charge. Free medical care is guaranteed for all visits to general practitioners, outpatient clinics, emergency departments, and public hospitals.

All Danish citizens are assigned a personal Civil Registration Number, which can be linked unambiguously to national registries on an individual level. Information on prior medical history can be retrieved from the National Patient Register. This register contains information on all inpatient activity at Danish hospitals since 1977 (and outpatient contacts since 1995) using International Classification of Diseases codes, revisions 8 and 10 (ICD-8 and ICD-10) [17]. Information on cause of death can be obtained from the Danish Register of Causes of Death, in which immediate, contributory, and underlying causes are recorded using ICD-10 codes [18]. This register is based on information from autopsy reports and death certificates. Causes of death is subsequently evaluated and corrected by the Danish Health and Medicines Authorities in case of obvious mistakes.

### Death certificates and forensic and hospital autopsy

Whenever a person dies in Denmark a death certificate is issued. The death certificate is always issued by a medical doctor, who on basis of all available information, including medical files, determines cause of death. Police involvement is mandatory in all suspicious deaths, deaths of unnatural manner, sudden and unexpected deaths, and if the deceased is found dead. In most cases, a medicolegal external examination is performed by the police and a Medical Doctor of Public Health who has access to first responder (emergency medical service) records, any medical files related to the deceased, the entire police reports including eye witness statements, and the body of the deceased. Information from all of these sources is collected in a *supplementary information field* on the death certificate, which makes Danish death certificates highly suitable for identification of sudden and unexpected death [10,11].

The final decision on whether to conduct a forensic autopsy is mandatorily made by the police, and autopsied cases include criminal cases and cases with unknown or uncertain manner of death. The forensic autopsy rate in Denmark is 2.3% of all deaths and 27% of deaths subjected to a medicolegal external examination [19]. The autopsy ratio among victims of sudden death in the 10-year study period was 61% [10,11]. Manner of death is natural in approximately 90% of forensic autopsy cases.

If it is decided not to conduct a forensic autopsy, physicians and relatives of the deceased can request a hospital autopsy. These are performed in local pathology departments throughout the country.

In both forensic and hospital autopsies a detailed macroscopic examination of the heart is performed. This is often followed by a microscopic cardiac examination, which includes a minimum of four and often more myocardial specimens sampled from different locations. The forensic autopsy also often includes additional examinations and tests such as histological examination of the conductive system, toxicology, biochemistry and microbiology. Molecular autopsy according to the existing guidelines is gradually incorporated as a diagnostic procedure, but has not been homogenously performed during the project period [20].

### Study population and data collection

We have previously identified all SCD in Denmark in individuals aged 1–35 years in 2000–2009 and 36–49 years in 2007–2009 [10,11]. All deaths within these age ranges in the 10-year period were included and all death certificates were reviewed independently by two physicians to identify cases of sudden and unexpected death. In cases of disagreement, the two investigators reevaluated the death certificate together to reach a consensus. Cases of sudden and unexpected death due to cardiac causes (SCD) were subsequently identified using autopsy reports, the Danish National Patient Register, and the Danish Register of Causes of Death together with access to discharge summaries and in selected cases medical records [10,11,21–24]. In case of uncertainty regarding cause of death after review of the autopsy report, the entire case in all its contents was reviewed by and discussed with a forensic cardio-pathologist [10,11].

### Definitions

The abbreviation SCD-myocarditis refers to a sudden cardiac death caused by myocarditis.

We defined *sudden death* as a sudden, natural, unexpected death; in witnessed cases, as an acute change in cardiovascular status with time to death being <1 h and, in unwitnessed cases, as a person last seen alive and functioning normally <24 h before being found death [10,11,25,26].

SCD in autopsied cases was defined as a sudden death of unknown or cardiac cause and in non-autopsied cases as a sudden death presumed to be of cardiac origin after review of all available information [10,11].

In autopsied SCD cases, a diagnosis of myocarditis required confirmation by histopathology according to the Dallas criteria which includes the presence of inflammatory infiltrates with/or without myocyte necrosis [27]. Positive toxicology was defined as the presence of any substance (licit and/or illicit) upon toxicological investigation.

### Statistical methods

Calculations and data analysis were performed with the use of statistical software (SAS^®^ 9.4; SAS Institute Inc., Cary, NC, USA). Differences in proportions were tested with the Fisher exact test. Continuous variables were compared using Wilcoxon test. Incidence rates of SCD-myocarditis were calculated as the ratio between SCD-myocarditis cases and person-years at risk. Person-years at risk were calculated using the mean resident population of Danes aged 1–35 years in 2000–2009 and 36–49 years in 2007–2009, as provided by Statistics Denmark [28]. Confidence intervals were calculated assuming Poisson distributed data. A two-sided *P*-value <0.05 was considered statistically significant.

## Results

### Study population

**Figure 1.**
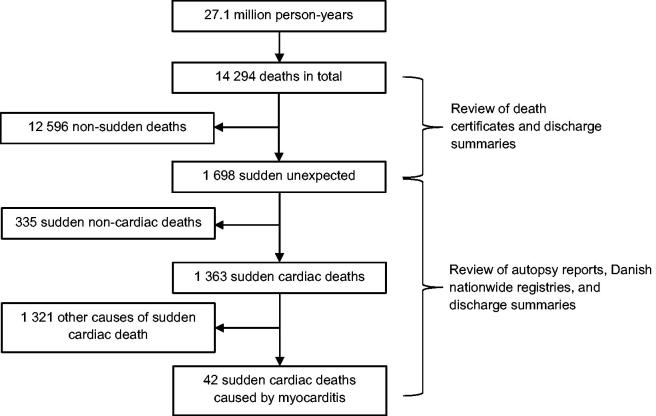
Flowchart of the process used to identify all cases of sudden cardiac death caused by myocarditis in persons aged 1–35 years and 1–49 years during 2000–2006 and 2007–2009, respectively.

The mean population of Danish residents aged 1–35 years in 2000–2009 and 36–49 years in 2007–2009 were 2.37 and 1.11 million, respectively. During the 10-year study period, there were 14 294 deaths from a total of 27.1 million person-years at risk (Figure 1). In total, 1 698 (12% of all deaths) cases of sudden and unexpected death were identified. After review of autopsy reports, death certificates, discharge summaries, and information from nationwide registries, 1 363 SCD (10% of all deaths) were identified, of which 753 (55%) were autopsied.

Cause of death was myocarditis in 42 SCD cases (3% of all SCD). All 42 SCD-myocarditis cases were autopsied (6% of autopsied SCD) and the diagnosis of myocarditis was confirmed through histopathological examination in all 42 cases. None of the nonautopsied SCD cases were deemed to be caused by myocarditis by the physician issuing the death certificate.

Of the 753 autopsies among all SCD cases, 620 (82%) were forensic autopsies and 133 (18%) were hospital autopsies. Among the 42 autopsied SCD-myocarditis cases, there were 41 (98%) forensic autopsies and one (2%) hospital autopsy.

A toxicology screen was performed in 477 (63%) of all 753 autopsied SCD, of which 270 (57%) had a positive toxicology. Of the 42 SCD-myocarditis cases, 25 (60%) received a toxicology screen, of which 14 (56%) had a positive toxicology. None of the toxicologically investigated SCD cases had a fatal toxicological profile. In two cases a toxic/allergic/autoimmune etiology was suspected: one case was started in clozapine treatment just prior to death, while the other case was diagnosed with polymyositis and cytotoxic T cells were identified in heart tissue through use of immunostaining. Between SCD-myocarditis cases and cases of SCD from other causes there were no significant differences in the proportion of cases with a toxicology screen or in the proportion of cases with positive toxicology (*P* = 0.623 and *P* = 0.950, respectively).

A postmortem virological examination on myocardial tissue specimens was performed in 10 (24%) of the 42 SCD-myocarditis cases with a positive result in three (30%) cases (two cases with parvovirus B19 and one case with missing information on virus type).

### Clinical characteristics

A comparison between autopsied SCD-myocarditis cases and cases with SCD from other causes is shown in Table 1. Among SCD-myocarditis cases median age at time of death was 31 years. Compared to cases with SCD from other causes, SCD-myocarditis cases were significantly younger at time of death (31 *vs.* 34 years, *P* = 0.027). A male predominance was seen in both SCD-myocarditis cases and cases of SCD from other causes with less than a third female cases (Table 1).

**Table 1.** Clinical characteristics and circumstances of cardiac arrest in autopsied cases of sudden cardiac death (SCD) caused by myocarditis and other causes in persons aged 1–35 years and 36–49 years in Denmark during 2000–2006 and 2007–2009, respectively.

Clinical characteristics and circumstances of cardiac arrest	SCD caused by myocarditis (*n* = 42)	SCD from other causes (*n* = 711)	*P-*value^a^
Median age, years (IQR)	31 (22–36)	34 (27–43)	0.027
Females	28 (22–36)	34 (26–43)	0.105
Males	33 (27–36)	34 (27–43)	0.110
Females, *n* (%)	13 (31)	212 (30)	0.864
Medicolegal external examination^b^, *n* (%)	40 (95)	606 (87)	0.150
Witnessed deaths^c^, *n* (%)	23 (56)	261 (39)	0.032
Previous medical history^d^, *n* (%)			
Psychiatric disease	8 (19)	142 (20)	0.884
Neurological disorder	4 (10)	73 (10)	0.877
Infectious disease	3 (7)	19 (3)	0.095
Cardiovascular disease	2 (5)	84 (12)	0.163
Ischemic heart disease	1 (2)	22 (3)	0.794
Heart failure	0 (0)	39 (5)	0.119
Cardiac dysrhythmia	0 (0)	32 (5)	0.160
Liver disease	2 (5)	17 (2)	0.341
Autoimmune disease	2 (5)	31 (4)	0.902
Diabetes mellitus	1 (2)	39 (5)	0.383
Cerebrovascular disease	0 (0)	21 (3)	0.259
Place of cardiac arrest, *n* (%)			
Home	25 (60)	459 (65)	0.575
Public place	11 (26)	164 (23)	
Hospital/ambulance	6 (14)	65 (9)	
Other	0 (0)	20 (3)	
Not specified	0 (0)	3 (0.4)	
Activity prior to cardiac arrest, *n* (%)			
Awake and relaxed	20 (48)	361 (51)	0.532
Sleep	18 (43)	236 (33)	
Physical activity	2 (5)	74 (10)	
Not specified	2 (5)	40 (6)	

a *P*-value for differences between SCD-myocarditis cases and cases with SCD from other causes.

b Data on whether a medicolegal external examination was performed were missing in 2% of all SCDs.

c Data on whether deaths were witnessed were missing in 5% of all SCDs.

d Treatment at hospital up to 5 years before death.

IQR: interquartile range.

The most common comorbidities among SCD-myocarditis cases were psychiatric diseases (19%), neurological disorders (10%), and infectious diseases (7%). Only two (5%) cases were diagnosed with cardiovascular disease prior to death. None of the SCD cases were previously diagnosed with myocarditis. There were no significant differences in frequency of any previously diagnosed medical conditions between SCD-myocarditis cases and cases with SCD from other causes. Frequency of reported infectious disease was, however, somewhat higher among SCD-myocarditis cases (7% *vs.* 3%, *P* = 0.095), although these changes did not reach statistical significance.

### Cardiac symptoms

A total of 20 (48%) SCD-myocarditis cases had at least one symptom prior to death (Table 2). The most common symptoms among SCD-myocarditis cases were influenza-like symptoms (*n* = 12, 29%), chest pain (*n* = 5, 12%), syncope (*n* = 3, 7%), and dyspnoea (*n* = 3, 7%). A comparison of symptom frequency among autopsied SCD-myocarditis cases and cases of SCD from other causes is shown in Table 2. The proportion of SCD-myocarditis cases with influenza-like symptoms reported in the days and weeks preceding death was ∼3-fold higher compared to cases with SCD from other causes (29% *vs.* 10%, *P* < 0.001). For the remaining symptom types there were no significant differences between the two groups.

**Table 2.** Symptoms prior to sudden cardiac death (SCD) in autopsied cases of SCD caused by myocarditis and other causes in persons aged 1–35 years and 36–49 years in Denmark during 2000–2006 and 2007–2009, respectively.

Symptoms prior to SCD^a^, *n* (%)	SCD caused by myocarditis (*n* = 42)	SCD from other causes (*n* = 711)	*P-*value^b^
Symptoms overall	20 (48)	302 (42)	0.513
Chest pain	5 (12)	127 (18)	0.324
Syncope	3 (7)	56 (8)	0.864
Dyspnoea	3 (7)	54 (8)	0.914
Seizures	2 (5)	12 (2)	0.152
Presyncope	1 (2)	16 (2)	0.957
Palpitations	1 (2)	7 (1)	0.550
Influenza-like symptoms	12 (29)	72 (10)	<0.001

a Symptoms were recorded within 1 year of death.

b *P*-value for differences between SCD-myocarditis cases and cases with SCD from other causes.

### Incidence rates

**Figure 2.**
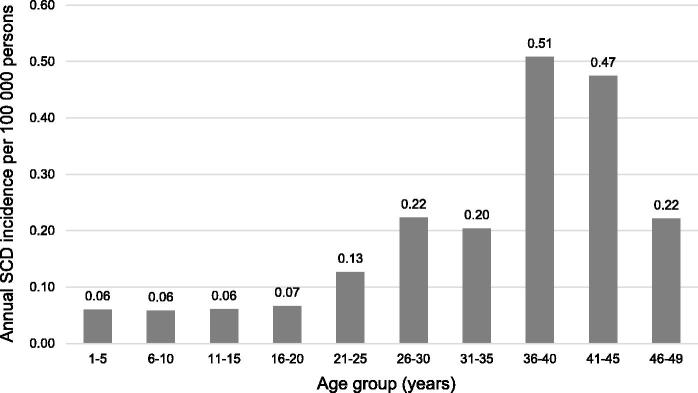
Age-related distribution of incidence of sudden cardiac death (SCD) caused by myocarditis per 100 000 person-years in persons aged 1–49 years in Denmark.

Incidence rates of SCD-myocarditis according to age and sex are shown in Table 3. There were low incidences of SCD-myocarditis among children and adolescents ranging from 0.06 to 0.07 per 100 000 person-years (Figure 2). Incidence of SCD-myocarditis increased sharply after the age of 20 years with the highest incidence (0.51 per 100 000 persons-years) observed in persons aged 36–40 years. Myocarditis was the cause of death in 6% (*n* = 42) of the 753 cases of autopsied SCD identified in the study period. However, the proportion of SCD caused by myocarditis was highly dependent on age: 8% in those aged 1–5 years, 18% in those aged 6–10 years, 7% in those aged 11–15 years, 5% in those aged 16–20 years, 7% in those aged 21–30 years, 6% in those aged 31–40 years, and 3% in those aged 41–49 years.

**Table 3.** Annual incidence rates of sudden cardiac death (SCD) caused by myocarditis per 100 000 person-years by age group and sex (95%CI).

Age group (years)	Total (*n* = 42)	Females (*n* = 13)	Males (*n* = 29)	*P-*value^a^
1–49	0.16 (0.11–0.21)	0.10 (0.06–0.17)	0.21 (0.15–0.30)	0.022
1–35	0.12 (0.08–0.17)	0.08 (0.04–0.15)	0.16 (0.10–0.25)	0.079
36–49	0.42 (0.25–0.71)	0.24 (0.09–0.65)	0.59 (0.32–1.10)	0.133

a *P*-value for difference between female and male cases of SCD caused by myocarditis.

**Figure 3.**
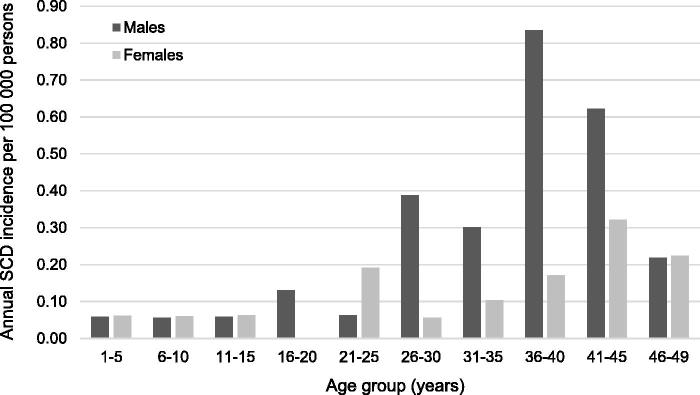
Sex-specific age-related distribution of incidence of sudden cardiac death (SCD) caused by myocarditis per 100 000 person-years in persons aged 1–49 years in Denmark.

Males had significantly higher incidence rates of SCD-myocarditis compared to females with an incidence rate ratio of 2.2 (95%CI: 1.1–4.1) (Table 3). Figure 3 indicates that the sex differences were more pronounced in the older age groups. There were, however, no significant differences in median age at time of death between female and male SCD-myocarditis cases (28 *vs.* 33 years, *P* = 0.401).

## Discussion

Myocarditis is a rare cardiac condition with an uncertain incidence in the general population. The proportion of SCD caused by myocarditis has been variably reported ranging from 1%–14% of all SCD [10–16]. The aim of this study was to establish SCD-myocarditis incidence among the young in a nationwide and unselected setting using validated and well-described definitions of SCD and myocarditis.

We found that the incidence of SCD-myocarditis was 0.16 per 100 000 person-years corresponding to 6% of autopsied SCD and 3% of all SCD, which is lower than estimates from multiple previous studies. Geographic differences may partially explain the dissimilarities in the previously reported incidences of SCD-myocarditis. However, use of different study populations and differences in SCD definition and classification and interpretation of myocardial inflammation are likely of greater importance. In a study by Kytö et al. [29], the death certificate-based incidence of fatal myocarditis in Finland was 0.46 per 100 000 person-years. This study, however, included persons of all ages and all myocarditis-related deaths and not only deaths presenting as SCD. Both incidence of myocarditis and SCD-myocarditis have previously been shown to be significantly higher among children and young adults compared to adults >50 years [29–31]. Surprisingly, in the present study highest SCD-myocarditis incidences were observed in those aged 36–45 years.

The SCD cohorts from which all SCD-myocarditis cases in this study were identified, did not include cases of sudden and unexpected death among infants less than 1 year of age as infants have an entirely different sudden death epidemiology compared to older children, adolescents, and young adults [23,32]. Therefore, in this study it was not possible to report SCD-myocarditis incidence among infants. However, previous studies report high incidences of myocarditis-related death in this age group [29,33]. Consequently, our reported SCD-myocarditis incidence would likely have been higher if infants were included, although this remains speculative.

When comparing SCD-myocarditis incidences between different studies it is important to address the diagnostic challenges subjected to myocarditis. Different approaches for the diagnosis of myocarditis can be utilized. Despite improvements in non-invasive diagnostic tests, histological examination remains gold standard for an unequivocal diagnosis of myocarditis in both living patients and the deceased. Several histologic criteria have been proposed from the Dallas criteria in 1987 to the latest quantitative criteria suggested by Maisch et al. [34], which has been incorporated in recent guidelines [3,20,27,34]. The Dallas criteria were made for endomyocardial biopsies and are subjected to several important limitations as being susceptible to variation in pathological interpretation and sampling error. In an autopsy setting, the unlimited accessibility to cardiac tissue can at least partly overcome the risk of sampling error, although the interpretation of the findings remains a challenge. Despite this the Dallas criteria are still widely used. A Finnish study examined how accurately myocarditis was diagnosed in a series of routine autopsies where cases recorded with myocarditis as the underlying cause of death in death certificates were re-examined histologically [35]. They found that only 32% of the re-examined cases met the Dallas criteria. This highlights that interobserver variability is a major problem and likely a contributory factor to the observed differences between reported SCD-myocarditis incidences.

Myocarditis has been reported to be overdiagnosed, especially in cases of sudden death with no other apparent explanation [35–37]. This may also be the case in our study as the diagnosis mainly was based on the Dallas criteria. The opposite, however, may also be a possibility. As the autopsy ratio among victims of sudden death in the examined age ranges was 61%, and since none of the non-autopsied cases had myocarditis reported as the cause of death, the incidence of SCD-myocarditis is very likely underestimated.

Viral infections are reported to underlie the majority of myocarditis cases in western countries and therefore virological analysis is recommended as a supplementary test to support a myocarditis diagnosis. In our study, virological examination was performed in 24% of the SCD-myocarditis cases with a positive result in 30% of these. The positive results may have contributed to a higher diagnostic accuracy in these cases. However, great caution should be paid when interpreting the results as the most commonly detected viruses (PVB-19, CMV, and HHV-6) can cause persistent infection with no or limited association to myocardial inflammation [38].

We found that 20 (48%) SCD-myocarditis cases had at least one symptom prior to SCD. The most common symptoms were influenza-like symptoms followed by chest pain, syncope, and dyspnoea. Influenza-like symptoms were significantly more common among SCD-myocarditis cases compared to cases with SCD from other causes, which supports the assumption of an infective agent as the most common etiology of myocarditis [4,39]. Although almost half of all SCD-myocarditis cases had symptoms prior to death, most had unspecific symptoms with limited diagnostic value. In line with this, none of the SCD-myocarditis cases were diagnosed with myocarditis prior to death. However, a subset of patients had syncope and seizures or symptoms suggestive of a cardiac etiology such as chest pains. These findings support the notion that young patients reporting potential cardiac symptoms should also be thoroughly examined.

Previous studies have shown that males have higher risk of SCD-myocarditis compared to females [29,40,41]. In support of this, we found that SCD-myocarditis incidence was >2-fold higher in males compared to females. Risk of SCD in general is known to be higher among men, despite similar risk profiles and causes of SCD in men and women [42]. This apparent female protection towards both SCD in general and SCD-myocarditis is poorly understood and warrants further investigation.

Psychiatric diseases were common among both SCD-myocarditis cases and cases of SCD from other causes with no significant differences. Psychiatric disease is a risk factor of both SCD and myocarditis. The elevated risk of SCD is, at least in part, mediated through unhealthy lifestyle and wide-spread use of medicine capable of inducing malignant cardiac arrhythmias among patients with psychiatric disease [43,44]. Some psychotropics, including clozapine, are known to cause toxic myocarditis [45,46]. Furthermore, alcohol misuse and use of recreational drugs, which is common in patients with severe psychiatric disease, can also cause myocarditis [47,48].

Toxicological analyses were performed in 60% of SCD-myocarditis cases and in 64% of cases with SCD from other causes with a positive result in tested cases of 56% and 57%, respectively. Between the two groups there were no significant differences in the proportion of cases with a toxicology screen or in the proportion of cases with positive toxicology, which is in contrast to results reported by Diaz et al. [49]. However, the groups in both studies are too small for detection of statistical differences.

### Strength and limitations

The main strength of our study is the nationwide and unselected approach with unselected inclusion and subsequent comprehensive follow-up of more than 14 000 deaths among the young in Denmark.

The study has limitations inherent to any retrospective study. However, the performance of a prospective study is complicated by the low incidence of SCD-myocarditis in the young general population.

The autopsy ratio among victims of sudden death in the 10-year study period was 61%. As mentioned previously, the selection of cases for forensic autopsy in Denmark relies on police investigations and the Danish legislation regarding the referral of individuals for medicolegal external examination and forensic autopsy. The selection of cases for hospital autopsies relies primarily on demands from the relatives. It follows that autopsy is not necessarily performed in all myocarditis-related deaths and consequently we likely underestimated the incidence of SCD-myocarditis.

As all SCD-myocarditis cases in this study were subjected to an autopsy the myocarditis diagnosis was mainly based on histological examination and in some cases supplementary tests such as virological examination and toxicological analysis. Given the high inter- and intraobserver variability related to the histological diagnosis, the study would have benefitted from a re-evaluation of the histological sections to secure that all cases fulfilled diagnostic criteria of myocarditis. However, 82% of all autopsies among SCD cases were forensic autopsies, which we consider a strength of the study as forensic autopsies in general include a more thorough examination with regard to supplementary analyses of the deceased.

Postmortem virological examination was only performed in 24% of all SCD-myocarditis cases. Systematic and thorough microbiological examination in all suspected myocarditis cases would strengthen the postmortem diagnosis of myocarditis

Our findings do not necessarily apply to other populations with other demographics as 90% of the study population were whites.

## Conclusion

In this nationwide study on SCD-myocarditis in children and young adults, we found a SCD-myocarditis incidence of 0.16 per 100 000 person-years corresponding to 6% of all autopsied SCD.

No cases of SCD-myocarditis were reported in the non-autopsied SCD cases, which could reflect underdiagnosing of myocarditis in non-autopsied SCD. As far from all cases of sudden and unexpected death is autopsied in both Denmark and internationally, the burden of SCD-myocarditis is likely underestimated. Our data highlight the importance of forensic autopsy in all cases of sudden and unexpected death in person <50 years to correctly establish cause of death.

Furthermore, our data suggest a female protection towards SCD-myocarditis. The underlying mechanism is poorly understood and warrants further investigation.

## References

[1] Sagar S, Liu PP, Cooper LT Myocarditis. Lancet 2012;379:738–747. 22185868 10.1016/S0140-6736(11)60648-X PMC5814111

[2] Caforio ALP, Malipiero G, Marcolongo R, et al. Myocarditis: a clinical overview. Curr Cardiol Rep. 2017;19:63. 28540649 10.1007/s11886-017-0870-x

[3] Caforio ALP, Pankuweit S, Arbustini E, et al. Current state of knowledge on aetiology, diagnosis, management, and therapy of myocarditis: a position statement of the European Society of Cardiology Working Group on Myocardial and Pericardial Diseases. Eur Heart J. 2013;34:2636–2648. 10.1093/eurheartj/eht210 23824828

[4] Hufnagel G, Pankuweit S, Richter A, et al. The European Study of Epidemiology and Treatment of Cardiac Inflammatory Diseases (ESETCID). First epidemiological results. Herz. 2000;25:279–285. 10904853 10.1007/s000590050021

[5] Kindermann I, Kindermann M, Kandolf R, et al. Predictors of outcome in patients with suspected myocarditis. Circulation. 2008;118:639–648. 18645053 10.1161/CIRCULATIONAHA.108.769489

[6] Mason JW Basic research on myocarditis: superb but unrequited. J Am Coll Cardiol. 2013;62:1746–1747. 23871883 10.1016/j.jacc.2013.06.030

[7] Richardson P, McKenna W, Bristow M, et al. Report of the 1995 World Health Organization/International Society and Federation of Cardiology Task Force on the definition and classification of cardiomyopathies. Circulation. 1996;93:841–842. 8598070 10.1161/01.cir.93.5.841

[8] Nippoldt TB, Edwards WD, Holmes DR, et al. Right ventricular endomyocardial biopsy: clinicopathologic correlates in 100 consecutive patients. Mayo Clin Proc. 1982;57:407–418. 6211578

[9] Fenoglio JJ, Ursell PC, Kellogg CF, et al. Diagnosis and classification of myocarditis by endomyocardial biopsy. N Engl J Med. 1983;308:12–18. 6860402 10.1056/NEJM198301063080103

[10] Winkel BG, Holst AG, Theilade J, et al. Nationwide study of sudden cardiac death in persons aged 1–35 years. Eur Heart J. 2011;32:983–990. 21131293 10.1093/eurheartj/ehq428

[11] Risgaard B, Winkel BG, Jabbari R, et al. Burden of sudden cardiac death in persons aged 1 to 49 years: nationwide study in Denmark. Circ Arrhythm Electrophysiol. 2014;7:205–211. 24604905 10.1161/CIRCEP.113.001421

[12] Doolan A, Langlois N, Semsarian C Causes of sudden cardiac death in young Australians. Med J Aust. 2004;180:110–112. 14748671 10.5694/j.1326-5377.2004.tb05830.x

[13] Subirana MT, Juan-Babot JO, Puig T, et al. Specific characteristics of sudden death in a mediterranean Spanish population. Am J Cardiol. 2011;107:622–627. 21184994 10.1016/j.amjcard.2010.10.028

[14] Bagnall RD, Weintraub RG, Ingles J, et al. A prospective study of sudden cardiac death among children and young adults. N Engl J Med. 2016;374:2441–2452. 27332903 10.1056/NEJMoa1510687

[15] Corrado D, Basso C, Rizzoli G, et al. Does sports activity enhance the risk of sudden death in adolescents and young adults? J Am Coll Cardiol. 2003;42:1959–1963. 14662259 10.1016/j.jacc.2003.03.002

[16] Wisten A, Krantz P, Stattin E-L Sudden cardiac death among the young in Sweden from 2000 to 2010: an autopsy-based study. Europace. 2017;19:1327–1334. 28873959 10.1093/europace/euw249

[17] Lynge E, Sandegaard JL, Rebolj M The Danish National Patient Register. Scand J Public Health. 2011;39:30–33. 21775347 10.1177/1403494811401482

[18] Helweg-Larsen K The Danish Register of Causes of Death. Scand J Public Health. 2011;39:26–29. 10.1177/1403494811399958 21775346

[19] Rapport from The National Police of Denmark [Internet] Available from: https://www.politi.dk/NR/rdonlyres/57C0A4A9-4B94-4197-8177-D321107B6206/0/EndeligRapport_arbejdsgruppenomdoedsfald.pdf [cited 19 September, 2018].

[20] Basso C, Aguilera B, Banner J, et al. Guidelines for autopsy investigation of sudden cardiac death: 2017 Update from the Association for European Cardiovascular Pathology. Virchows Arch. 2017;471:691–705. 28889247 10.1007/s00428-017-2221-0 PMC5711979

[21] Lynge TH, Risgaard B, Jabbari R, et al. Cardiac symptoms before sudden cardiac death caused by hypertrophic cardiomyopathy: a nationwide study among the young in Denmark. Europace. 2016;18:1801–1808. 26823388 10.1093/europace/euv403

[22] Glinge C, Jabbari R, Risgaard B, et al. Symptoms before sudden arrhythmic death syndrome: a nationwide study among the young in Denmark. J Cardiovasc Electrophysiol. 2015;26:761–767. 25807988 10.1111/jce.12674

[23] Lynge TH, Jeppesen AG, Winkel BG, et al. Nationwide study of sudden cardiac death in people with congenital heart defects aged 0 to 35 years. Circ Arrhythm Electrophysiol. 2018;11:e005757. 29858381 10.1161/CIRCEP.117.005757

[24] Sadjadieh G, Jabbari R, Risgaard B, et al. Nationwide (Denmark) study of symptoms preceding sudden death due to arrhythmogenic right ventricular cardiomyopathy. Am J Cardiol. 2014;113:1250–1254. 24513468 10.1016/j.amjcard.2013.12.038

[25] Kong MH, Fonarow GC, Peterson ED, et al. Systematic review of the incidence of sudden cardiac death in the United States. J Am Coll Cardiol. 2011;57:794–801. 21310315 10.1016/j.jacc.2010.09.064 PMC3612019

[26] Myerburg C Cardiac arrest and sudden cardiac death In: Zipes DP, Libby P, Bonow RO, et al., editors. Braunwald’s heart disease. A textbook of cardiovascular medicine. Philadelphia (PA): Elsevier Saunders; 2007 p. 933–973.

[27] Aretz HT Myocarditis: the Dallas criteria. Hum Pathol. 1987;18:619–624. 3297992 10.1016/s0046-8177(87)80363-5

[28] Statistics Denmark [Internet]. Available from: http://www.dst.dk/en [cited 17 August, 2018].

[29] Kytö V, Saraste A, Voipio-Pulkki L-M, et al. Incidence of fatal myocarditis: a population-based study in Finland. Am J Epidemiol. 2007;165:570–574. 17237135 10.1093/aje/kwk076

[30] Li L, Zhang Y, Burke A, et al. Demographic, clinical and pathological features of sudden deaths due to myocarditis: results from a state-wide population-based autopsy study. Forensic Sci Int. 2017;272:81–86. 28122325 10.1016/j.forsciint.2016.12.037

[31] Kytö V, Sipilä J, Rautava P The effects of gender and age on occurrence of clinically suspected myocarditis in adulthood. Heart 2013;99:1681–1684. 24064227 10.1136/heartjnl-2013-304449

[32] Winkel BG, Holst AG, Theilade J, et al. Sudden unexpected death in infancy in Denmark. Scand Cardiovasc J. 2011;45:14–20. 21133644 10.3109/14017431.2010.538433

[33] Webber SA, Boyle GJ, Jaffe R, et al. Role of right ventricular endomyocardial biopsy in infants and children with suspected or possible myocarditis. Br Heart J. 1994;72:360–363. 7833195 10.1136/hrt.72.4.360 PMC1025547

[34] Maisch B, Portig I, Ristic A, et al. Definition of inflammatory cardiomyopathy (myocarditis): on the way to consensus. A status report. Herz. 2000;25:200–209. 10904839 10.1007/s000590050007

[35] Kytö V, Saukko P, Lignitz E, et al. Diagnosis and presentation of fatal myocarditis. Hum Pathol. 2005;36:1003–1007. 16153464 10.1016/j.humpath.2005.07.009

[36] Hauck AJ, Kearney DL, Edwards WD Evaluation of postmortem endomyocardial biopsy specimens from 38 patients with lymphocytic myocarditis: implications for role of sampling error. Mayo Clin Proc. 1989;64:1235–1245. 2593714 10.1016/s0025-6196(12)61286-5

[37] Chow LH, Radio SJ, Sears TD, et al. Insensitivity of right ventricular endomyocardial biopsy in the diagnosis of myocarditis. J Am Coll Cardiol. 1989;14:915–920. 2794278 10.1016/0735-1097(89)90465-8

[38] Nielsen TS, Hansen J, Nielsen LP, et al. The presence of enterovirus, adenovirus, and parvovirus B19 in myocardial tissue samples from autopsies: an evaluation of their frequencies in deceased individuals with myocarditis and in non-inflamed control hearts. Forensic Sci Med Pathol. 2014;10:344–350. 24781135 10.1007/s12024-014-9570-7

[39] Bowles NE, Ni J, Kearney DL, et al. Detection of viruses in myocardial tissues by polymerase chain reaction. Evidence of adenovirus as a common cause of myocarditis in children and adults. J Am Coll Cardiol. 2003;42:466–472. 12906974 10.1016/s0735-1097(03)00648-x

[40] Wang H, Yao Q, Zhu S, et al. The autopsy study of 553 cases of sudden cardiac death in Chinese adults. Heart Vessels 2014;29:486–495. 23836068 10.1007/s00380-013-0388-0

[41] Mason JW, O'Connell JB, Herskowitz A, et al. A clinical trial of immunosuppressive therapy for myocarditis. The Myocarditis Treatment Trial Investigators. N Engl J Med. 1995;333:269–275. 7596370 10.1056/NEJM199508033330501

[42] Winkel BG, Risgaard B, Bjune T, et al. Gender differences in sudden cardiac death in the young—A nationwide study. BMC Cardiovasc Disord. 2017;17:19. doi: 10.1186/s12872-016-0446-5 PMC5219679 28061807

[43] Risgaard B, Waagstein K, Winkel BG, et al. Sudden cardiac death in young adults with previous hospital-based psychiatric inpatient and outpatient treatment: a nationwide cohort study from Denmark. J Clin Psychiatry. 2015;76:1122–1129. 10.4088/JCP.14m09742 26455676

[44] Timour Q, Frassati D, Descotes J, et al. Sudden death of cardiac origin and psychotropic drugs. Front Pharmacol. 2012;3:76. doi: 10.3389/fphar.2012.00076 22590457 PMC3349287

[45] Coulter DM, Bate A, Meyboom RH, et al. Antipsychotic drugs and heart muscle disorder in international pharmacovigilance: Data mining study. Br Med J. 2001;322:1207–1209. 11358771 10.1136/bmj.322.7296.1207 PMC31617

[46] Yuen JWY, Kim DD, Procyshyn RM, et al. Clozapine-induced cardiovascular side effects and autonomic dysfunction: a systematic review. Front Neurosci. 2018;12:203. doi: 10.3389/fnins.2018.00203 29670504 PMC5893810

[47] Kloner RA, Hale S, Alker K, et al. The effects of acute and chronic cocaine use on the heart. Circulation. 1992;85:407–419. 1346509 10.1161/01.cir.85.2.407

[48] Rezkalla SH, Hale S, Kloner RA Cocaine-induced heart diseases. Am Heart J. 1990;120:1403–1408. 2248185 10.1016/0002-8703(90)90255-v

[49] Diaz FJ, Loewe C, Jackson A Death caused by myocarditis in Wayne County, Michigan: A 9-year retrospective study. Am J Forensic Med Pathol. 2006;27:300–303. 17133024 10.1097/01.paf.0000221045.67949.6e

